# Editorial: Navigating environmental hazards in the workplace: impacts and interventions

**DOI:** 10.3389/fpubh.2026.1893964

**Published:** 2026-06-26

**Authors:** Sasho Stoleski, Dragan Mijakoski, Milan Milošević, Hana Brborović

**Affiliations:** 1 Faculty of Medicine, Institute of Occupational Health of R. North Macedonia, WHO CC, GA2LEN CC, Ss. Cyril and Methodius, University in Skopje, Skopje, North Macedonia; 2 Department of Environmental and Occupational Health and Sports Medicine, Andrija Štampar School of Public Health, University of Zagreb, School of Medicine, Zagreb, Croatia

**Keywords:** environmental hazard assessment, occupational health and safety, occupational medicine, organizational culture, psychosocial hazards, workplace interventions, workplace safety and health

## Introduction

The nature of occupational health hazards has evolved considerably over the past century. Traditional occupational health frameworks were largely concerned with acute physical injuries, industrial accidents, and exposure to toxic substances in manufacturing and heavy industry. However, the modern workplace presents a far more complex landscape of environmental, psychosocial, ergonomic, organizational, and technological risks. Today's workers are simultaneously exposed to multiple interacting hazards that collectively influence physical health, psychological wellbeing, productivity, organizational resilience, and long-term quality of life. This complexity is reflected in the diverse contributions included in this Research Topic. For example, Małysa and Chrapoński demonstrate how data-driven statistical approaches can support workplace safety improvement, while Nyarubeli et al. illustrate the continuing burden of traditional physical exposures through their investigation of whole-body vibration and chronic low back pain among transport and heavy-equipment workers.

The Research Topic “*Navigating Environmental Hazards in the Workplace: Impacts and Interventions*,” published in *Frontiers in Public Health*, contains 46 manuscripts authored by 264 researchers and provides a multidisciplinary perspective on contemporary occupational health challenges. The collection addresses a broad spectrum of workplace hazards, including ergonomic strain, vibration exposure, psychosocial stress, fatigue, occupational noise, asbestos exposure, organizational burnout, and intervention strategies aimed at improving worker safety and resilience. Importantly, the Research Topic reflects the growing recognition that occupational health can no longer be viewed solely through biomedical or regulatory perspectives. Instead, workplace wellbeing emerges from the interaction of environmental conditions, organizational systems, worker behavior, social determinants, and broader societal transformations. This broader perspective is particularly evident in studies such as the longitudinal work of Jerg-Bretzke et al., which demonstrated how effort–reward imbalance and work–family conflict influence perceptions of patient safety, and in Schulte's framework for addressing climate-related occupational hazards, which highlights the expanding scope of occupational health research and policy.

The collection is particularly timely given the unprecedented changes affecting global labor markets and workplace environments. Rapid technological transformation, digitalization, globalization, climate change, aging populations, hybrid work models, and post-pandemic organizational restructuring have all reshaped occupational risk profiles. Workers increasingly face chronic and cumulative exposures rather than isolated hazards. Mental health concerns, burnout, fatigue, and psychosocial stress have become as relevant to occupational safety as chemical exposure and physical injury. Pinheiro et al., for example, demonstrate that occupational health outcomes extend well beyond traditional workplace accidents by examining non-cancer mortality among firefighters, while Jansen et al. show how behavioral and organizational factors influence the use of hearing protection devices in occupational environments. At the same time, Schulte's climate framework, together with findings from Idris et al. on hydration-related health outcomes among construction workers and the policy analysis by Kathayat et al., illustrate how environmental change is emerging as a critical determinant of occupational health.

The articles included in this Research Topic collectively demonstrate that workplace hazards are multidimensional and interconnected. Physical and psychological risks frequently coexist and reinforce one another. Organizational culture, management practices, workload distribution, social support, and worker participation all influence the effectiveness of occupational health interventions. Importantly, the collection moves beyond merely documenting workplace hazards and instead emphasizes preventive strategies, intervention frameworks, and integrated approaches to worker protection. Odawara et al. demonstrate how workplace tobacco-control programs can be adapted while maintaining implementation fidelity, whereas Dilek Kart and Meydanlioglu show that targeted educational interventions can improve knowledge, preventive behaviors, and low back pain awareness among marble workers. Together, these studies reinforce the importance of translating occupational health evidence into practical workplace solutions.

This editorial synthesizes the major themes emerging from the Research Topic while exploring their broader implications for occupational health policy, organizational management, workplace sustainability, and future research directions. Drawing upon contributions ranging from climate-related occupational hazards and psychosocial health to fatigue prediction and occupational disease surveillance, the editorial highlights how contemporary occupational health increasingly depends on integrated approaches capable of addressing the complex realities of modern work environments.

## The changing landscape of occupational environmental hazards

Historically, occupational environmental hazards were primarily associated with industrial settings involving hazardous machinery, chemical agents, and unsafe physical working conditions. While such hazards remain highly relevant, modern workplaces increasingly expose employees to more complex combinations of physical, ergonomic, psychosocial, and organizational risks. The studies included in this Research Topic clearly demonstrate this transition and illustrate how contemporary occupational health challenges arise from the interaction of multiple workplace determinants.

Several contributions focus on traditional occupational hazards, including vibration exposure, asbestos exposure, occupational noise, and chemical risks. At the same time, an equally important proportion of the collection addresses psychosocial stressors, burnout, fatigue, work–family conflict, and organizational dynamics. This dual focus reflects a growing understanding that occupational health outcomes are shaped by cumulative and interacting exposures rather than isolated environmental factors. Zhu et al., for example, explore the evolving nature of occupational hazards in urban settings, while Obeidat et al. demonstrate how environmental conditions such as temperature extremes may directly influence physical performance and functional capacity.

One of the key contributions within the collection examines whole-body vibration exposure among heavy machinery operators and truck drivers. Nyarubeli et al. reported a strong association between prolonged vibration exposure and chronic low back pain among Tanzanian workers, highlighting the continued burden of occupational musculoskeletal disorders despite decades of ergonomic research and preventive recommendations. These findings reinforce concerns regarding prolonged driving, inadequate seat suspension systems, poor road conditions, repetitive mechanical stress, and insufficient ergonomic protections. Similar themes emerge in studies examining physically demanding occupations, including those of beach workers and other labor-intensive sectors, where cumulative mechanical loading continues to influence long-term musculoskeletal health.

Importantly, the Tanzanian study also illustrates occupational health inequities across global regions. Workers in low- and middle-income countries frequently experience greater exposure levels, fewer regulatory protections, and more limited access to occupational healthcare services. As highlighted by Schulte's broader framework for emerging occupational risks, preventive approaches must account for differences in economic development and infrastructure if global occupational health goals are to be achieved. Similar concerns arise in the work of Zhang et al., whose investigation of benzene exposure among healthcare workers demonstrates that occupational hazards remain relevant even within sectors not traditionally considered highly exposed. Furthermore, Dou et al. identified that archivists are exposed to significant occupational hazards spanning chemical, biological, physical, and ergonomic dimensions, which contribute to a range of health issues. These findings underscore the necessity for in-depth research into archivists' occupational health and the urgent development of targeted protective strategies to address these hazards.

The importance of prevention is further reinforced by the study of Dilek Kart and Meydanlioglu, which demonstrated that structured ergonomic training programs improved knowledge, preventive behaviors, and safety practices among marble workers. Their findings support a central principle of occupational health: effective hazard control requires not only engineering interventions and regulatory compliance but also worker engagement, occupational health literacy, and continuous education. This perspective is echoed by Zhou and Chen and by Zhou W. et al., whose work on musculoskeletal disorders among older hospital cleaners highlights the value of awareness, risk recognition, and targeted preventive measures in reducing occupational health burdens. Moreover, Saik et al. conducted a study aimed to develop a process for determining a set of alternative preventive measures to reduce risk levels, using the example of reducing the incidence of occupational pneumoconiosis in miners under conditions of financial cost minimization. A key feature of this study is the improvement in the risk management process by integrating the efficiency factor of risk reduction under financial constraints.

## Ergonomic risks and musculoskeletal disorders

Musculoskeletal disorders (MSDs) remain among the most prevalent occupational health conditions worldwide and continue to represent a major source of disability, reduced productivity, absenteeism, and diminished quality of life. Despite decades of research and the implementation of numerous ergonomic interventions, the studies included in this Research Topic demonstrate that MSDs remain a persistent challenge across diverse occupational sectors. Collectively, these contributions highlight the complex interplay between physical workload, workplace design, organizational conditions, worker behavior, and aging-related vulnerabilities.

A recurring finding throughout the collection is the continuing importance of biomechanical exposures in shaping musculoskeletal health outcomes. Nyarubeli et al. demonstrated a significant association between whole-body vibration exposure and chronic low back pain among heavy-equipment operators and truck drivers, illustrating how prolonged exposure to mechanical stress can contribute to chronic occupational disease. Similar concerns emerge in studies involving construction workers, beach workers, and healthcare support staff, where repetitive motion, awkward postures, prolonged standing, and manual material handling continue to place workers at increased risk of musculoskeletal injury.

The systematic review conducted by Santos et al. provides particularly compelling evidence of the global burden of work-related musculoskeletal disorders among construction workers. Their findings reveal consistently high prevalence rates across multiple regions and occupational settings, suggesting that despite improvements in workplace safety regulations, many workers continue to experience exposures capable of producing long-term musculoskeletal damage. Likewise, Sousa dos Santos et al. demonstrate that physically demanding occupations outside traditional industrial settings are similarly affected, reinforcing the notion that ergonomic risks are not confined to a single sector or occupation.

The studies included in this Research Topic also emphasize the cumulative nature of musculoskeletal risk. Occupational exposures rarely occur in isolation. Instead, repetitive movements, forceful exertion, prolonged physical loading, inadequate recovery periods, and organizational pressures often interact over extended periods. This perspective is particularly evident in the work of Zhou S. et al., who examined musculoskeletal disorders among older hospital cleaners. Their findings highlight the additional challenges faced by aging workforces and demonstrate how long-term occupational exposures may interact with age-related physiological changes to increase vulnerability to injury and disability.

Importantly, the collection moves beyond describing ergonomic risks and places considerable emphasis on prevention. Dilek Kart and Meydanlioglu demonstrated that educational interventions can significantly improve workers' knowledge and preventive behaviors related to low back pain. Their findings suggest that occupational health literacy and worker engagement represent important complements to engineering controls and ergonomic redesign. This perspective is further supported by Feng et al., whose development of a low back pain assessment tool for island workers illustrates the importance of context-specific surveillance and early identification strategies.

A broader population perspective is provided by Zhao et al., who examined global trends in low back pain and neck pain among working populations. Their analysis confirms that musculoskeletal disorders remain among the leading causes of occupational disability worldwide despite substantial advances in prevention and treatment. These findings reinforce the need for sustained investment in workplace ergonomics, worker education, occupational surveillance, and integrated prevention programs.

Collectively, the studies addressing musculoskeletal health suggest that effective prevention requires a multidimensional approach that extends beyond workplace design alone. Engineering controls, worker training, health literacy, organizational support, exposure monitoring, and early intervention strategies all contribute to reducing the burden of musculoskeletal disorders. As work environments continue to evolve, integrated ergonomic programs will remain a cornerstone of occupational health practice.

## Occupational noise, hearing protection, and safety behavior

Occupational noise exposure remains one of the most widespread environmental hazards encountered across modern workplaces. Although noise-induced hearing loss has long been recognized as a major occupational disease, the studies included in this Research Topic demonstrate that the consequences of occupational noise extend far beyond auditory health. Noise influences communication, concentration, fatigue, cognitive performance, workplace behavior, and overall safety culture.

The continuing public health significance of occupational noise is illustrated by Gong (a) et al., who examined the global burden of occupational noise-induced hearing loss. Their findings demonstrate that hearing impairment attributable to workplace noise remains a substantial health challenge worldwide and is likely to continue affecting large numbers of workers in the coming decades. Such evidence underscores the importance of maintaining effective hearing conservation programs even as occupational environments become increasingly automated and technologically advanced.

Importantly, the Research Topic demonstrates that occupational noise is not restricted to traditionally noisy industrial sectors. Torres-Cobo et al. examined occupational noise exposure within university environments and highlighted how noise can affect workers in settings that are not commonly associated with hearing-related hazards. Their findings reinforce the need for broader recognition of occupational noise as a cross-sectoral workplace issue.

The effects of noise also extend beyond hearing loss itself. Ke et al. demonstrated that environmental noise exposure can influence workplace behavior and performance, suggesting that noise should be considered within broader occupational safety frameworks. Reduced concentration, impaired communication, increased fatigue, and diminished situational awareness may all contribute to elevated workplace risk. Consequently, noise exposure should be viewed not only as an environmental hazard but also as a factor capable of influencing human performance and organizational outcomes.

Several studies within the collection further demonstrate the importance of organizational influences on protective behavior. Jansen et al. explored hearing protection device use from the perspectives of employees, supervisors, and safety managers, revealing that compliance is strongly influenced by workplace culture, communication practices, and leadership commitment. Their findings suggest that successful hearing conservation programs depend not only on the availability of protective equipment but also on organizational environments that encourage and support its consistent use.

This observation aligns closely with broader findings related to safety climate and occupational health literacy. Nisar et al. demonstrated that safety behavior mediates the relationship between safety climate and workplace outcomes, emphasizing the importance of organizational commitment to safety. Similarly, Zhou S. et al. highlighted the role of occupational health literacy in promoting protective workplace behaviors. Together, these studies suggest that sustainable reductions in occupational noise-related harm require a combination of engineering controls, worker education, organizational support, and behavioral interventions.

The evidence presented throughout the collection therefore supports a comprehensive approach to noise management. Protecting workers from occupational noise requires more than exposure monitoring and hearing protection devices. It also requires organizational cultures that prioritize prevention, encourage worker participation, and integrate safety considerations into everyday workplace practice.

## Psychosocial hazards and workplace mental health

One of the most prominent themes emerging from this Research Topic is the growing importance of psychosocial hazards as determinants of occupational health. Contemporary workplaces increasingly expose workers to organizational pressures, emotional demands, workload challenges, fatigue, and work–life conflicts that can significantly affect both individual wellbeing and organizational performance.

The studies included in this Research Topic demonstrate that psychosocial risks are not confined to specific professions but are evident across a wide range of occupational groups. Healthcare workers, government employees, nurses, seafarers, professional drivers, and other worker populations all experience psychosocial stressors capable of influencing health outcomes, productivity, safety, and quality of care.

Jerg-Bretzke et al. provide a particularly important contribution through their longitudinal investigation of effort–reward imbalance and work–family conflict. Their findings demonstrate that psychosocial stress can influence perceptions of patient safety, illustrating how worker wellbeing and organizational outcomes are closely interconnected. Rather than affecting employees alone, psychosocial hazards may have broader implications for service quality, workplace performance, and organizational effectiveness.

Similar themes emerge in the work of Wu and Liang, who examined the relationship between psychological contracts and burnout among government employees. Their findings suggest that employees' perceptions of fairness, reciprocity, and organizational commitment play an important role in shaping mental health outcomes. When workers perceive a mismatch between expectations and organizational realities, burnout and disengagement may become more likely.

Fatigue represents another recurring concern throughout the collection. Qu et al. developed a fatigue prediction model among healthcare workers, demonstrating the potential value of proactive approaches for identifying individuals at increased risk of adverse occupational outcomes. Likewise, Ma and Liao's review of occupational fatigue among seafarers highlights the multifactorial nature of fatigue, including workload, sleep disruption, organizational conditions, and environmental influences.

The consequences of fatigue are particularly evident in the work of Palandri et al., who reported associations between sleepiness, occupational accidents, and near misses among professional drivers. Their findings reinforce the notion that fatigue is not simply a personal health issue but a significant occupational safety concern with implications for injury prevention and workplace performance.

Psychological wellbeing is also influenced by workplace support systems. Dziedzic et al. demonstrated the protective role of social support among nurses experiencing occupational stress, emphasizing the importance of workplace relationships and supportive organizational cultures. Similarly, Song et al. identified differences in anxiety and sleep quality among anesthesiologists working in different subspecialties, highlighting how occupational context may shape psychological outcomes even within the same profession.

Taken together, these studies illustrate that psychosocial hazards are now central components of occupational health. Effective prevention strategies must therefore extend beyond traditional hazard control measures and incorporate organizational interventions, leadership development, workload management, social support systems, and mental health promotion programs capable of addressing the broader determinants of worker wellbeing.

## Organizational culture and psychological contracts

While physical, chemical, and environmental exposures remain central concerns in occupational health, the studies included in this Research Topic consistently demonstrate that organizational environments play an equally important role in shaping worker wellbeing and safety outcomes. Increasingly, occupational health research recognizes that workplace culture, leadership practices, communication systems, organizational justice, and employee engagement influence not only mental health but also productivity, safety behavior, and organizational resilience.

A key contribution to this discussion is provided by Wu and Liang, who examined the relationship between psychological contracts and burnout among government employees. Their findings suggest that workers' perceptions of fairness, reciprocity, and organizational commitment are critical determinants of psychological wellbeing. When employees perceive that organizational obligations have not been fulfilled, trust may erode, resulting in emotional exhaustion, disengagement, and burnout. These findings reinforce the idea that occupational health cannot be separated from broader organizational dynamics.

The importance of organizational conditions is further illustrated by the work of Jerg-Bretzke et al., who demonstrated that effort–reward imbalance contributes to work–family conflict and influences perceptions of patient safety. Their findings extend the discussion beyond individual health outcomes and highlight how organizational structures may ultimately affect service quality, patient care, and operational performance. Together, these studies suggest that worker wellbeing and organizational effectiveness are closely interconnected and should not be viewed as separate objectives.

Supportive workplace relationships also emerge as important protective factors. Dziedzic et al. demonstrated that social support among nurses plays a significant role in mitigating occupational stress and enhancing psychological resilience. Their findings highlight the value of supportive leadership, collaborative work environments, and positive interpersonal relationships in helping workers cope with occupational demands. Such evidence suggests that workplace culture can serve either as a protective factor or as a source of additional stress depending on how organizations are managed.

The influence of organizational culture on safety outcomes is particularly evident in the work of Nisar et al., who demonstrated that safety behavior mediates the relationship between safety climate and workplace outcomes. Their findings indicate that workers are more likely to engage in protective behaviors when safety is perceived as an organizational priority. Rather than relying solely on compliance-based approaches, effective occupational health programs require cultures in which safe practices are actively encouraged, reinforced, and supported by leadership.

Additional contributions within the collection emphasize the role of organizational learning and occupational health literacy. Bexten et al. examined educational approaches addressing the second victim phenomenon among healthcare professionals, demonstrating how targeted interventions can strengthen organizational support systems and improve worker wellbeing. Similarly, Zhou S. et al. highlighted the importance of occupational health literacy among essential workers, illustrating how knowledge, awareness, and risk perception influence workplace behavior and preventive practices.

Collectively, these studies demonstrate that organizational culture should be considered a core determinant of occupational health. Leadership quality, communication practices, worker participation, organizational justice, and social support all influence employees' ability to recognize risks, engage in preventive behaviors, and maintain long-term wellbeing. Consequently, future occupational health strategies must address organizational environments alongside traditional workplace hazards.

## Legacy hazards and long-term occupational disease

Although emerging psychosocial, technological, and environmental challenges are increasingly shaping occupational health research, the studies included in this Research Topic demonstrate that traditional occupational exposures continue to impose a substantial burden of disease worldwide. Occupational cancers, respiratory diseases, and chronic exposure-related conditions remain important public health concerns, particularly because their effects often become apparent many years after the original exposure has occurred.

The continuing burden of asbestos-related disease provides a striking example of this challenge. Chen et al. documented trends in cancers attributable to occupational asbestos exposure in China, highlighting the long-term consequences of exposures that may have occurred decades earlier. Their findings illustrate one of the defining characteristics of occupational disease: the latency period between exposure and disease manifestation can be extremely long, meaning that workers continue to experience adverse health outcomes long after exposure controls have been introduced.

A similar pattern emerges in studies addressing occupationally related cancers. Mandanach et al. reviewed evidence linking occupational exposures to bladder cancer, while Gong (b) et al. and Wang D. et al. examined the burden of laryngeal cancer associated with workplace carcinogens such as asbestos and sulfuric acid. Together, these studies demonstrate that occupational carcinogens remain significant contributors to global disease burden despite advances in industrial hygiene and occupational safety regulation. Their findings reinforce the importance of long-term surveillance, exposure monitoring, and preventive strategies designed to minimize cumulative workplace exposures.

The collection also highlights the continuing relevance of chemical hazards beyond well-known carcinogens. Zhang et al. identified benzene exposure risks among healthcare workers, illustrating that occupational chemical exposures may occur in sectors not traditionally viewed as highly hazardous. Similarly, Duan et al. documented the burden associated with occupational formaldehyde exposure, demonstrating how evolving industrial processes and workplace environments continue to create new exposure scenarios. These studies remind us that occupational health vigilance must extend beyond historically recognized high-risk industries.

Respiratory disease represents another important theme within the collection. Stoleski et al. examined chronic obstructive pulmonary disease among working populations and highlighted the continuing impact of occupational exposures on respiratory health. Their findings are complemented by the work of Wang X.-P. et al., who investigated psychological symptoms among patients with pneumoconiosis. Together, these studies illustrate that occupational diseases often affect far more than physical health alone. Chronic respiratory conditions may influence quality of life, psychological wellbeing, social functioning, and long-term work ability, emphasizing the need for holistic approaches to worker health.

The healthcare sector also remains vulnerable to occupational exposures. Karkaz et al. documented sharps injuries and splash exposures among healthcare personnel, while Shen et al. and Wang L. et al. examined blood borne occupational exposures and intervention strategies designed to reduce risk. Although these exposures differ from chronic environmental hazards such as asbestos and benzene, they similarly demonstrate the importance of effective prevention systems and organizational commitment to worker safety.

Taken together, these studies illustrate that traditional occupational hazards remain highly relevant despite shifts toward newer occupational health challenges. The burden of occupational disease reflects the cumulative effects of environmental exposures, workplace practices, regulatory systems, and organizational decisions operating over extended periods. Consequently, effective occupational health strategies must balance attention to emerging risks with continued efforts to prevent and monitor long-standing occupational diseases.

## Climate change and emerging occupational risks

Climate change is increasingly recognized as one of the most significant emerging challenges facing occupational health systems worldwide. While occupational health has traditionally focused on hazards originating within the workplace itself, environmental changes occurring at regional and global levels are creating new exposure patterns that directly affect worker health, safety, and productivity.

A conceptual framework for understanding these challenges is provided by Schulte, who argues that climate-related occupational hazards require dedicated research agendas and policy responses. His work highlights the numerous pathways through which environmental change may influence occupational exposures, including rising temperatures, extreme weather events, altered air quality, and changes in the distribution of biological hazards. Importantly, this framework emphasizes that climate-related risks are already affecting workplaces rather than representing distant future concerns.

Evidence supporting this perspective is provided by Idris et al., who examined hydration-related health outcomes among construction workers. Their findings demonstrate that heat exposure and hydration challenges are already influencing worker health in physically demanding outdoor occupations. As global temperatures continue to rise, such risks are likely to become increasingly important across sectors including construction, agriculture, transportation, mining, and emergency response.

The impact of environmental conditions on occupational performance is further illustrated by Obeidat et al., who investigated upper-limb function under temperature extremes. Their findings suggest that environmental exposures may directly affect physical performance and workplace safety, providing evidence that climate-related occupational risks extend beyond heat illness alone. Environmental conditions may influence dexterity, reaction time, fatigue, and overall work capacity, with important implications for injury prevention and operational effectiveness.

The broader health implications of environmental change are explored by Lang et al., who proposed a gender-integrated bio-psychosocial model for understanding cardiovascular risk associated with environmental hazards. Their work highlights the importance of considering individual susceptibility, social determinants, and occupational context when evaluating environmental risks. Such perspectives are particularly important as occupational health systems seek to address increasingly diverse worker populations exposed to changing environmental conditions.

Policy preparedness represents another important theme within the collection. Kathayat et al. compared occupational safety and health risk-management policies across different settings and demonstrated considerable variation in preparedness for emerging occupational challenges. Their findings suggest that climate resilience will require not only scientific understanding but also adaptive regulatory frameworks, workplace preparedness plans, and coordinated public health responses.

Collectively, these studies indicate that climate change is expanding the scope of occupational health beyond traditional workplace boundaries. Future occupational health strategies will increasingly need to incorporate environmental monitoring, climate adaptation measures, emergency preparedness, and resilience planning. As environmental conditions continue to evolve, occupational health systems must adapt accordingly to ensure the protection of workers across diverse occupational settings.

## Technology, digitalization, and new occupational challenges

Technological transformation is reshaping workplaces at an unprecedented pace, creating both opportunities and challenges for occupational health. Advances in digitalization, automation, predictive analytics, and data-driven decision-making have expanded the tools available for workplace risk assessment and prevention. At the same time, these developments have introduced new concerns related to cognitive workload, fatigue, human–technology interaction, and the changing nature of occupational risk itself.

Several studies included in this Research Topic illustrate how technological innovation can support occupational health management. Qu et al. developed a predictive model for identifying fatigue among healthcare workers, demonstrating how data-driven approaches can facilitate early risk detection and proactive intervention. Similarly, Małysa and Chrapoński highlighted the value of statistical methods in workplace safety management, showing how analytical approaches can strengthen evidence-based decision-making and improve the effectiveness of prevention programs. Together, these studies suggest that occupational health is increasingly moving from reactive responses toward predictive and preventive strategies.

Despite these advances, technological development does not eliminate traditional occupational health challenges. The collection demonstrates that fatigue remains a significant concern across multiple sectors. Ma and Liao's review of occupational fatigue among seafarers emphasizes the complex interactions among workload, organizational factors, environmental conditions, and sleep disruption. Similar concerns emerge in the work of Song et al., who identified differences in anxiety and sleep quality among anesthesiologists, and Palandri et al., who reported associations between sleepiness, occupational accidents, and near misses among professional drivers. These findings indicate that even in technologically advanced workplaces, human performance remains influenced by biological and psychosocial factors that cannot be fully addressed through technology alone.

Emerging evidence also suggests that workplace exposures may influence cognitive functioning through complex pathways. Li et al. examined interactions between occupational hazards and genetic susceptibility in relation to cognitive performance among aluminum workers. Their findings highlight the growing importance of interdisciplinary research that integrates occupational medicine, neuroscience, genetics, and public health. Such approaches may become increasingly relevant as occupational health seeks to understand how environmental and workplace factors influence cognitive outcomes over the course of working life.

Technological innovation also offers significant opportunities for future occupational health practice. Wearable sensors, real-time exposure monitoring systems, predictive analytics, artificial intelligence, and digital health platforms may enable earlier identification of hazards and more individualized intervention strategies. These technologies have the potential to improve surveillance, enhance risk assessment, and support evidence-based workplace decision-making.

However, technological transformation also raises important questions regarding privacy, surveillance, worker autonomy, and ethical governance. Increased digital connectivity and data collection may contribute to new forms of occupational stress if not implemented appropriately. Consequently, technological innovation should be viewed neither as a universal solution nor as a new source of risk alone. Rather, it represents an evolving context within which occupational health systems must operate, requiring careful consideration of both opportunities and challenges.

Collectively, the studies included in this Research Topic suggest that the future of occupational health will depend on the successful integration of technological innovation with worker-centered approaches that prioritize health, safety, wellbeing, and organizational sustainability.

## Toward integrated occupational health frameworks

A central message emerging from this Research Topic is that occupational health can no longer be effectively addressed through fragmented approaches that consider physical, psychological, environmental, and organizational risks separately. The studies included in this Research Topic consistently demonstrate that worker health outcomes arise from interactions among multiple determinants operating simultaneously within complex workplace systems.

The diversity of contributions illustrates this multidimensional reality. Studies addressing musculoskeletal disorders highlight the influence of physical workload, ergonomic design, worker behavior, and aging-related vulnerabilities. Research examining occupational noise demonstrates that environmental exposures affect not only physiological outcomes but also concentration, communication, and safety performance. Investigations into burnout, fatigue, occupational stress, and psychological contracts reveal the importance of organizational environments in shaping worker wellbeing. Meanwhile, studies of occupational cancers, respiratory diseases, and chemical exposures remind us that traditional environmental hazards continue to generate long-term health consequences that require sustained preventive efforts.

Taken together, these findings suggest that occupational health is best understood through a systems perspective. Physical hazards may influence psychological wellbeing, psychosocial stress may affect safety behavior, organizational culture may determine compliance with preventive measures, and environmental conditions may interact with individual susceptibility to shape health outcomes. Such complexity challenges traditional occupational health models focused on single exposures and supports the development of integrated frameworks capable of addressing multiple interacting determinants simultaneously.

This perspective aligns with contemporary approaches such as Total Worker Health, organizational resilience models, and sustainable workplace frameworks. These approaches emphasize prevention, worker participation, interdisciplinary collaboration, and long-term health promotion rather than narrow compliance-based strategies. Several studies included in the Research Topic support these principles directly. Research addressing occupational health literacy, safety climate, worker training, tobacco-control implementation, and intervention effectiveness demonstrates that sustainable improvements depend as much on organizational commitment and worker engagement as on technical controls.

The collection also highlights the importance of interdisciplinary collaboration. Addressing contemporary occupational health challenges requires expertise from occupational medicine, epidemiology, psychology, ergonomics, engineering, environmental science, public health, organizational behavior, and data analytics. As workplaces become increasingly complex, collaboration across disciplines will be essential for developing effective and sustainable interventions.

Ultimately, the studies included in this Research Topic reinforce the idea that occupational health should be viewed not simply as the prevention of injury and disease but as the promotion of healthy, resilient, and sustainable work environments capable of supporting workers throughout their careers.

## Future directions for occupational health research and policy

The findings presented throughout this Research Topic provide valuable insights into the challenges currently facing occupational health systems while simultaneously identifying important priorities for future research, policy development, and workplace practice.

One of the most urgent priorities involves integrating climate change considerations into occupational health frameworks. Evidence presented by Schulte, Idris et al., Obeidat et al., and Lang et al. demonstrates that environmental change is already influencing workplace conditions, exposure patterns, and health outcomes. Future research should focus on identifying vulnerable worker populations, evaluating adaptation strategies, and developing climate-resilient occupational health policies capable of responding to emerging environmental risks.

Mental health represents a second major priority. Burnout, fatigue, work–family conflict, occupational stress, anxiety, and sleep disturbances emerge repeatedly throughout the collection as important determinants of worker wellbeing and organizational performance. Studies by Jerg-Bretzke et al., Wu and Liang, Qu et al., Song et al., Palandri et al., and Dziedzic et al. collectively demonstrate the need for proactive organizational interventions capable of addressing psychosocial hazards before adverse outcomes occur. Future workplace programs should therefore focus on prevention, resilience building, leadership development, and organizational support systems.

Technological transformation represents another important area for future investigation. The work of Małysa and Chrapoński, Qu et al., and Li et al. illustrates both the opportunities and challenges associated with digitalization and predictive analytics. Future research should explore how artificial intelligence, wearable technologies, real-time monitoring systems, and digital health platforms can improve occupational health outcomes while simultaneously addressing concerns related to privacy, worker autonomy, and ethical governance.

The collection also highlights persistent occupational health inequities across occupations, industries, and geographic regions. Studies involving workers in low-resource settings demonstrate that exposure levels, preventive resources, and access to occupational healthcare remain unevenly distributed. Future policies should therefore prioritize vulnerable worker populations and strengthen occupational health infrastructure globally.

Another important research priority involves understanding cumulative and interacting exposures across the working lifespan. Many occupational hazards do not occur in isolation but emerge through interactions among environmental, organizational, psychosocial, and individual factors. Longitudinal research, systems-based methodologies, and interdisciplinary approaches will therefore be increasingly important for understanding occupational health outcomes and informing preventive strategies.

Taken together, the findings presented throughout this Research Topic suggest that the future of occupational health lies in adaptive, integrated, and worker-centered approaches capable of addressing the complexity of modern work environments while remaining responsive to emerging societal and environmental challenges.

## Conclusion

The Research Topic “Navigating Environmental Hazards in the Workplace: Impacts and Interventions” provides a comprehensive and timely contribution to contemporary occupational health research. Collectively, the 46 manuscripts demonstrate that occupational hazards are increasingly multidimensional, arising through interactions among environmental exposures, organizational conditions, worker behaviors, technological systems, and broader societal changes.

Several overarching themes emerge from the collection. First, traditional occupational hazards remain highly relevant. Musculoskeletal disorders, occupational noise, chemical exposures, respiratory diseases, and occupational cancers continue to impose substantial burdens across diverse industries and occupations. At the same time, psychosocial hazards, burnout, fatigue, organizational stressors, and mental health concerns have become central components of occupational health discourse, reflecting the changing nature of work in the 21st century.

Second, the collection highlights the growing importance of organizational factors in shaping occupational outcomes. Safety climate, occupational health literacy, leadership practices, social support, psychological contracts, and worker participation consistently emerge as important determinants of both health and safety. These findings suggest that effective occupational health strategies must address organizational environments alongside physical hazards.

Third, the studies demonstrate that emerging challenges such as climate change, technological transformation, and demographic shifts are reshaping occupational risk profiles. Addressing these challenges will require occupational health systems that are flexible, interdisciplinary, and capable of adapting to rapidly changing workplace conditions.

Perhaps most importantly, the Research Topic emphasizes that occupational health should be viewed through an integrated lens. Worker wellbeing cannot be reduced solely to the prevention of injury or disease. Rather, it reflects the combined influence of physical, psychological, organizational, environmental, and social determinants operating throughout the working lifespan. Protecting worker health therefore requires comprehensive strategies that combine hazard control, health promotion, organizational development, education, and evidence-based policy.

By bringing together evidence from diverse occupational settings and research traditions, this Research Topic advances understanding of both established and emerging workplace hazards while highlighting opportunities for intervention and prevention. The collection ultimately serves not only as a scientific contribution but also as a call to action for researchers, employers, policymakers, and occupational health professionals. As workplaces continue to evolve, occupational health systems must evolve with them, embracing holistic, adaptive, and worker-centered approaches capable of promoting healthy, safe, and sustainable work environments for future generations.

